# Classical conditioning of Purkinje cell responses *in vitro* produces *in vivo*-like simple spike suppressions during the conditional stimulus

**DOI:** 10.1016/j.bbrep.2026.102493

**Published:** 2026-02-06

**Authors:** Artem Gornov, Thiago C. Moulin, Fredrik Johansson

**Affiliations:** Department of Experimental Medical Science, Lund University, Lund, Sweden

## Abstract

An *in vitro* model of classical conditioning could improve our understanding of underlying mechanisms thanks to controlled pharmacological manipulation and negligeable perturbations from external networks. Here, using mice cerebellar slices and patch clamp recordings, we present a protocol for classical conditioning of Purkinje cells *in vitro* at room temperature. Repeated pairing of a parallel fiber conditional stimulus and a climbing fiber unconditional stimulus with parameters compatible with *in vivo* learning gives rise to a Purkinje cell simple spike suppression during the conditional stimulus that shares similarities with conditioned responses observed in live animals.

## Highlights

• New approach to classical conditioning *in vitro* with parameters used *in vivo*.
• Circumvents *in vitro* limitation, with precise control on concentration and delivery of pharmacological agent.
• Blocking GIRK channels *in vitro* prevents learning similarly to *in vivo* studies.

Classical eyeblink conditioning is one of the simplest experimental tasks of associative learning that depends upon the cerebellar cortex [[1], [2], [3], [4]]. It consists of successive presentations of a behaviorally neutral conditional stimulus (CS) followed, after a fixed temporal delay, by an unconditional stimulus (US) that elicits an initial unconditioned response, the eyeblink. After a variable amount of time and paired CS-US presentations called trials, the subject develops a conditioned response (CR) to the previously neutral CS, consisting of an eyeblink peaking near the anticipated onset of the US. Replacing a behavioral CS with direct stimulation of the mossy fibers and a behavioral US with direct stimulation of the climbing fibers (cf) or of the inferior olive, produced reliable CRs in both rabbits [5] and ferrets [6]. The overt blinks are driven by learned simple spike suppressions during CS presentation (or “pause” responses) of Purkinje cells (PCs) in physiologically defined cortical zones [[7], [8], [9]].

The leading hypotheses for how this learning occurs are based on conventional synaptic plasticity with timing arising from time-varying activation of different parallel fibers (pf) [[10], [11], [12]]. In contrast, nonconventional ‘cell-intrinsic’ mechanisms have also been suggested, based on the finding that when the CS consists of direct activation of the pf *in vivo*, PCs acquire timed conditioned responses [13]. A large number of studies conducted *in vitro* ([[14], [15], [16], [17]], and see Ref. [18] for an extensive review) use stimulation parameters mostly incompatible with learning of a conditioned response *in vivo*, due to non-characteristic inter-trial intervals, insufficiently long CS-US intervals[19], insufficient cf stimulation pulses [20] or a combination of those limitations. Furthermore, more recent studies using parameters compatible with learning *in vivo* [21], focus on long-term depression (LTD) induction as an outcome and not simple spike suppressions as in behavioral classical conditioning *per se*. To complete them and further understand the mechanisms underlying classical conditioning, we applied parameters exactly as used *in vivo* to an *in vitro* classical conditioning protocol. This allows us to verify whether the simple spike suppressions during pf stimulation observed *in vivo* and associated with behavioral learning can also be observed *in vitro*.

In the following experiments, a partially reinforced classical conditioning protocol alternating between paired and unpaired trials was applied to 45 Purkinje cells recorded in cell-attached patch clamp from parasagittal cerebellar slices (vermis and paravermis) from male mice (P21-52), without discrimination between lobules. As the CS, we used pf stimulation (200 ms, 100Hz) delivered via two electrodes positioned in a way that allows to stimulate separate parallel fiber bundles passing through the distal and medial parts of the dendritic tree. Burst cf stimulation (10 ms, 500Hz) applied to the PC's sole climbing fiber was used as the US. These parameters were chosen to match the ones used *in vivo* that reliably produce CRs [13]. The conditioning protocol consisted of an alternation between a paired stimulation, the first electrode delivering the pf stimulation followed by the cf stimulation resulting in a 200 ms CS-US interval; and an unpaired stimulation, the second electrode delivering the pf stimulation not followed by a cf stimulation, with an intertrial interval of 15 s. The effective interval between consecutive paired pc + cf stimulations was thus 30 s. The conditioning protocol lasted as long as allowed by the stability of the recording, up to 780 trials (∼6h of recording) with a median of 160 paired trials (80min). All the following analysis is limited to the paired stimulations of the protocol. The results of the paired pf + cf protocol are compared to results from cells recorded in identical conditions with the cf stimulation turned off, serving as a control.

The data set includes both Purkinje cells that were silent and that spontaneously fired at rates up to ∼40 Hz. In about half of the population (n = 21) circulating artificial cerebrospinal fluid (composition in mM: NaCl 120; KCl 2; CaCl_2_ 2; NaHCO_3_ 26; MgSO_4_ 1.19; KH_2_PO_4_ 1.18; d-glucose 11 at pH 7.4 continuously equilibrated with a 5% CO_2_ in O_2_ mix. Internal solution composition, in mM: KMeSO3 135; NaCl 4; KCl 10; ethylene glycol-bis(β-aminoethyl ether)-N,N,N′,N'-tetraacetic acid 1; 4-(2-hydroxyethyl)-1-piperazineethanesulphonic acid 10; NaGTP 0.4 and MgATP 2 at pH 7.35 adjusted with KOH) was heated to 34 °C. The rest of the cells (n = 24) were recorded at room temperature. 10 of those cells were recorded in presence of 5 μM Tertiapin LQ (TLQ), a high affinity and high specificity inhibitor of G protein-coupled inwardly rectifying potassium channels (GIRK 1-4 also called K_ir_ 3.1/4) [22] that affects conditioning *in vivo* [23]. This concentration was chosen to maximize the binding to Kir3.x channels (c > 10K_d_), while minimizing the binding to any other potential target channel (c < 0.5K_d_ of the second most binding receptor).

**Fig. 1:**
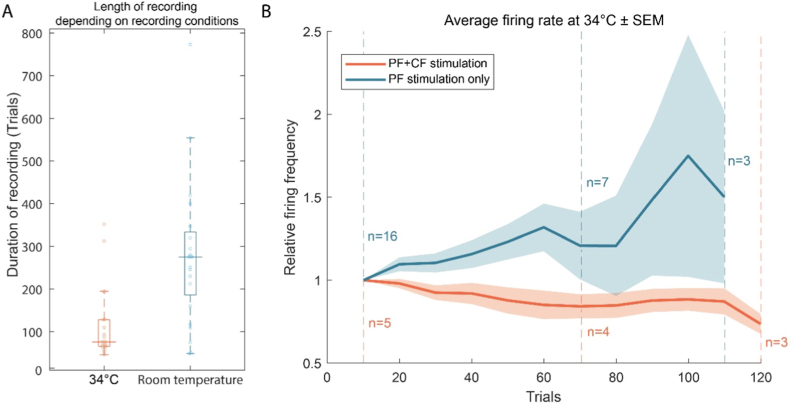
**Purkinje cell behavior in 34°C recording conditions.** (a) Boxplots comparing the durations of recordings of PCs at 34 °C and at room temperature. (b) Average firing rate of PCs, only at 34 °C, during pf stimulationrelative to the baseline response (first ten trials) in PF + CF stimulation (orange) and PF stimulation only (blue) conditions. Vertical dashed lines indicate the number of cells still being recorded and included in the average calculation at a given number of trials. Curves are interrupted when the population of cells recorded for the indicated number of trials falls below 3.

Recordings carried out at 34 °C did not remain stable as long as recordings at room temperature (Mann-Whitney *U* test, p = 3.4∗10^−4^, n = 45) with more than half the recordings (n = 12) not stable enough to reach 80 paired trials (Fig. 1a) which is most likely insufficient to observe clear signs of learning [8]. Purkinje cells received either only the parallel fiber CS (“PF stimulation only” trials; 16/21 cells) or additionally received a burst climbing fiber US at the end of the pf stimulation (“PF + CF stimulation” trials; 5/21 cells). We chose to characterize and quantify PC behavior by calculating average firing rates for every cell during pf stimulation by blocks of 10 trials, relative to the first 10 trials that established the baseline response (Fig. 1b). The average firing frequency of PCs during pf stimulation seems to decrease over time with the conditioning protocol but remains stable or even increases if no cf stimulation is paired with the pf stimulation. Due to the insufficient length of recordings, all further investigations have been carried out at room temperature.

**Fig. 2:**
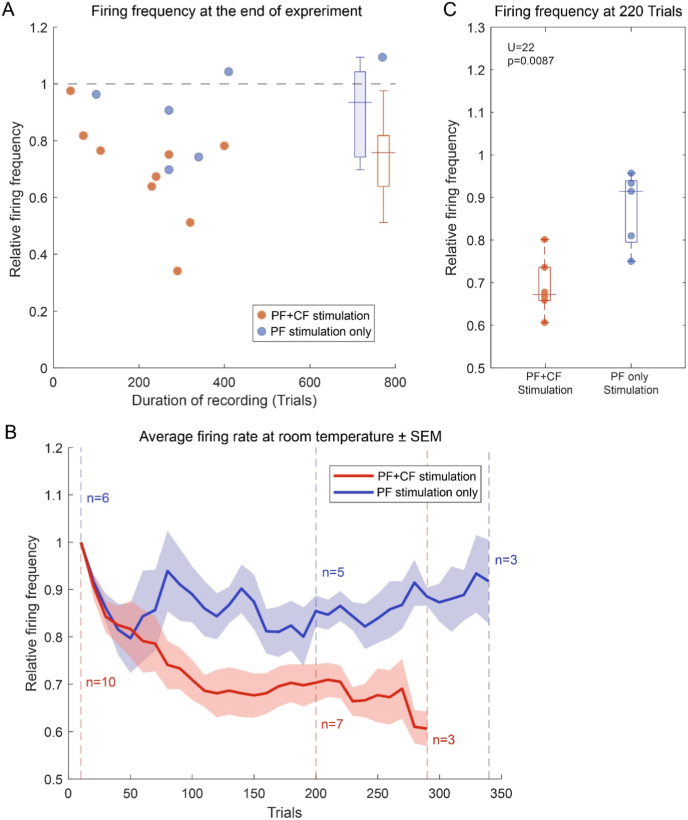
**Purkinje cells decrease their firing rate during pf stimulation only with PF + CF stimulation.** (a) Population plot of the change in firing rate during pf stimulation (n = 16, the baseline response compared to the last 10 trials) for PF stimulation only (blue) and PF + CF stimulation (red) conditions. Each circle is an individual Purkinje cell and the circle location on the x-axis indicates the duration of that particular experiment in number of trials. Dotted horizontal line represents the baseline firing rate during pf stimulation. Boxplots show the median, the interquartile range and the extremes (b) Average firing rate of PCs during pf stimulation relative to the baseline response in PF + CF stimulation (red) and PF stimulation only (blue) conditions. As in Fig. 1 vertical dotted lines quantify the cells still being recorded and curves are interrupted when the population of cells recorded for the indicated number of trials falls below 3. (c) Boxplot comparing firing frequency of individual cells during pf stimulation 220 trials into the recording relative to the beginning of the recording for PF + CF stimulation (red) and PF stimulation only (blue) conditions. Boxplots show the median, quartiles, minimum, maximum and all individual cells.

At room temperature the recording length increased (median = 275 paired trials) to resemble *in vivo* experiments and allow better understanding of the *in vitro* response of PCs to either repeated pf only or pf + cf stimulation. We first aimed to establish whether paired parallel fiber-climbing fiber stimulation in a classical conditioning protocol causes reductions in simple spike responses during pf stimulation. PCs responses were unaffected by pf only trials (Fig. 2). A Wilcoxon paired signed rank test comparing the firing at baseline and at the end of recording failed to show a difference (p = 0.56, n = 6). Furthermore, 220 trials into the recording all cells still being recorded failed to show a significant decrease compared to their baseline firing rate in response to pf stimulation only (Wilcoxon paired signed rank test, p = 0.0625, n = 5, Fig. 2b) though a slight reduction in firing rate cannot be excluded. In contrast paired pf + cf stimulation trials caused a significant decrease in firing frequency during pf stimulation both at the end of recordings (Wilcoxon paired signed rank test, p = 0.002, n = 10) and 220 trials into the recording for all remaining cells (Wilcoxon paired signed rank test, p = 0.0313, n = 6). After 220 trials, the cells exposed to paired pf + cf stimulation conditions, fired significantly less during pf stimulation compared to cells exposed to pf stimulations only (Mann-Whitney *U* test, p = 0.0087, n = 12, Fig. 2c).

**Fig. 3:**
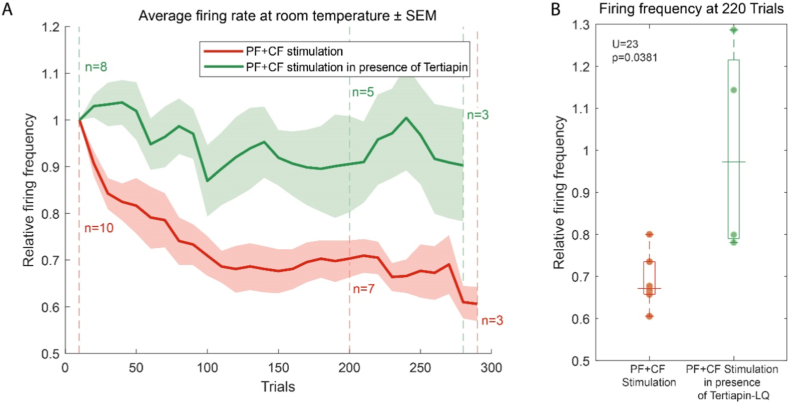
**Tertiapin-LQ prevents the decrease in firing rate during pf stimulation.** (a) Average firing rate of PCs during the pf stimulation relative to the baseline response in presence (green) and absence (red) of Tertiapin-LQ. As in Fig. 1 vertical dotted lines quantify the cells still being recorded and curves are interrupted when the population of cells recorded for the indicated number of trials falls below 3. (b) Boxplot comparing firing frequency of individual cells during pf stimulation 220 trials into the recording relative to the beginning of the recording, in absence (red) and presence (green) of Tertiapin. Boxplots show the median, quartiles, minimum, maximum and all individual cells.

As a further validation of the *in vitro* model, we also applied one of the few pharmacological agents that has shown an effect *in vivo*. The pf + cf protocol was run in presence of TLQ. Addition of TLQ to the bath did not impact the spontaneous firing rate of PCs, their baseline response to pf stimulation or their ability to elicit a complex spike in response to cf stimulation. The cells were recorded in a bath containing enough TLQ to suppress >95% of all K_ir_ channels and <5% of any other potassium channel. Learning during the protocol was impaired. The cells presented no evidence for differences in firing rate during pf stimulation (Fig. 3) at the end of the recordings (Wilcoxon paired signed rank test, p = 0.742, n = 8). For all cells recorded in presence of TLQ for at least 220 trials, we observed no change in firing rate during pf stimulation at 220 trials compared to their baseline response (Wilcoxon paired signed rank test, p = 0.625, n = 5). At 220 trials, cells being recorded in presence of TLQ differed significantly in firing frequency during pf stimulation compared to cells recorded without any pharmacological intervention (Mann-Whitney *U* test, p = 0.038, n = 12, Fig. 3b). Overall, the dynamics observed in the presence of TLQ are almost identical to the behavior observed in PCs exposed to pf stimulation only.

There have been many studies of classical conditioning involving cerebellar learning *in vivo*. Here we show that a classical conditioning protocol with a long CS and a burst US as used *in vivo* also seems to work *in vitro* to circumvent some of the limitations of *in vivo* recordings. Applying this protocol *in vitro* causes substantial reductions in simple spike responses to the pf stimulation. One of the main benefits of this protocol is precise control over the quantities of pharmacological agents delivered to the PC during the recording, which we used to apply a Kir3.x antagonist TLQ, that has shown to be effective to suppress the conditioned response when applied after conditioning *in vivo* despite uncertainty on delivery concentration.

While Purkinje cells consistently acquired a simple spike suppression in protocols using both pf and cf stimulation, the extent of this effect was variable. There are several plausible reasons for this.

The experiment durations were variable and often lasted much less than the duration of analogous *in vivo* experiments. For unknown reasons, *in vivo* conditioning requires a variable number of trials, but in general many more trials than the current experiments reached, to produce the characteristic ‘conditioned Purkinje cell responses' [8]. As the experiments at 34 °C were not lasting long enough, we decided to conduct recordings at room temperature in order to improve the duration of recording. This affects the physiology of the PC and preliminary observations tend to show a smaller fraction of PCs exhibiting spontaneous firing in a room temperature setting, and a lower firing rate in the PCs that do fire spontaneously. The recording durations got significantly longer than in warm bath conditions, but that positive might be offset by a longer learning period. It is nonetheless interesting to note that learning curves in both recording conditions produce similar dynamics (magnitude and rate of learning) of simple spike suppression in Purkinje cells exposed to the conditioning protocol, at the durations shared by both conditions. This supports the idea that the learning mechanism is conserved at lower temperatures. The temperature can also influence plasticity conditions in the PC, the extent of which has not been measured.

Another concern is the spread of learning mechanisms throughout the cerebellum as the zonal identity of PCs in our data is not known. Nevertheless, the learning displayed by PCs in the experimental conditions is consistent with learning curves *in vivo* [24] and it is therefore convincing that paired parallel fiber CS-climbing fiber US trials cause a substantial reduction in simple spike responses to the pf stimulation. If the zonal identity was deeply relevant, we deem it unlikely to observe such consistent effects.

The protocol used was different from most classical conditioning protocols as between each paired trial, another stimulation is delivered via a second electrode situated at the same depth but at a different distance to the soma than the one delivering the paired stimulation. One of the electrodes could therefore serve as a control for the unpaired response in the same cell. Post hoc tests on different cells have shown some interactions between both electrodes and since it has likely happened on some recordings. It is impossible to know which recordings have been affected, preventing analysis of the unpaired trials. In absence of interaction the protocol is equivalent to a classical conditioning protocol with a 30 s intertrial interval. For the recordings where the interaction is potentially present the protocol is equivalent to a 50% reinforced classical conditioning with 15 s intertrial interval. While it has been shown to slow the rate of learning [25,26], it does not prevent it, and all cells recorded longer than a 110 trials show a decrease in firing rate during pf stimulation superior to 20%.

We also evaluated PC conditioned pause response timing to see if it is compatible with results *in vivo*. Latency to the maximum firing suppression should be adaptively timed to the duration of the CS-US interval [13,24]. In this data set we did not observe such adaptive timing. It is possible that the duration of the recordings can explain failure to observe a properly timed response.

Lastly, we also show that the application of K_ir_3 antagonist Tertiapin_LQ_ during conditioning prevents the expression of the learned response, as previously observed *in vivo*. Further experiments with TLQ could allow for better understanding of the mechanism by which it suppresses Purkinje cell learning, by, for example, establishing a dose-response curve. The scope of this paper does not allow us to infer further on the exact mechanism underlying the observed learning. Kir3.x could be activated by different sources including mGluR7 and GABAB receptors and spike frequency modulation in the PC is a complex phenomenon, that can be driven by LTD, non-LTD synaptic mechanisms and PC-intrinsic mechanisms. Nevertheless, the findings suggest that this method of classical conditioning *in vitro* can contribute to *in vivo* studies where controlled drug delivery has been problematic.

In conclusion, our data shows the possibility of observing learning in a Purkinje cell *in vitro* by applying a classical conditioning protocol, with *in vivo* parameters, stimulating parallel fibers and climbing fibers as CS and US, respectively.

## Funding

This work was supported by grants to FJ from the 10.13039/501100004359Swedish Research Council (2019-02034), the 10.13039/501100001725Royal Swedish Academy of Sciences (ME2019-0048), the 10.13039/501100000942Swedish Brain Foundation (FO2020-0005), the Craaford Foundation (20200529 as well as the Åke Wiberg, Magnus Bergvall and Segerfalk foundations.

## CRediT authorship contribution statement

**Artem Gornov:** Conceptualization, Formal analysis, Investigation, Writing – original draft. **Thiago C. Moulin:** Investigation. **Fredrik Johansson:** Conceptualization, Funding acquisition, Methodology, Supervision, Writing – review & editing.

## Declaration of competing interest

The authors declare that they have no known competing financial interests or personal relationships that could have appeared to influence the work reported in this paper.

## Data Availability

Data will be made available on request.

## References

[1] Hardiman M.J., Ramnani N., Yeo C.H. (1996). Reversible inactivations of the cerebellum with muscimol prevent the acquisition and extinction of conditioned nictitating membrane responses in the rabbit. Exp. Brain Res..

[2] Yeo C.H., Hardiman M.J., Glickstein M. (1985). Classical conditioning of the nictitating membrane response of the rabbit. I. Lesions of the cerebellar nuclei. Exp. Brain Res..

[3] Yeo C.H., Hardiman M.J., Glickstein M. (1985). Classical conditioning of the nictitating membrane response of the rabbit. II. Lesions of the cerebellar cortex. Exp. Brain Res..

[4] Yeo C.H., Hardiman M.J., Glickstein M. (1985). Classical conditioning of the nictitating membrane response of the rabbit. III. Connections of cerebellar lobule HVI. Exp. Brain Res..

[5] Steinmetz J.E., Lavond D.G., Thompson R.F. (1989). Classical conditioning in rabbits using pontine nucleus stimulation as a conditioned stimulus and inferior olive stimulation as an unconditioned stimulus. Synapse.

[6] Hesslow G., Svensson P., Ivarsson M. (1999). Learned movements elicited by direct stimulation of cerebellar mossy fiber afferents. Neuron.

[7] Heiney S.A., Kim J., Augustine G.J., Medina J.F. (2014). Precise control of movement kinematics by optogenetic inhibition of Purkinje cell activity. J. Neurosci..

[8] Jirenhed D.A., Hesslow G. (2016). Are Purkinje cell pauses drivers of classically conditioned blink responses?. Cerebellum.

[9] Ten Brinke M.M., Boele H.J., Spanke J.K., Potters J.W., Kornysheva K., Wulff P., Ac I.J., Koekkoek S.K., De Zeeuw C.I. (2015). Evolving models of Pavlovian conditioning: cerebellar cortical dynamics in awake behaving mice. Cell Rep..

[10] Lepora N.F., Porrill J., Yeo C.H., Dean P. (2010). Sensory prediction or motor control? Application of marr-albus type models of cerebellar function to classical conditioning. Front. Comput. Neurosci..

[11] Medina J.F., Mauk M.D. (2000). Computer simulation of cerebellar information processing. Nat. Neurosci..

[12] Yamazaki T., Tanaka S. (2009). Computational models of timing mechanisms in the cerebellar granular layer. Cerebellum.

[13] Johansson F., Jirenhed D.A., Rasmussen A., Zucca R., Hesslow G. (2014). Memory trace and timing mechanism localized to cerebellar Purkinje cells. Proc. Natl. Acad. Sci. U. S. A..

[14] Crepel F., Jaillard D. (1991). Pairing of pre- and postsynaptic activities in cerebellar Purkinje cells induces long-term changes in synaptic efficacy in vitro. J. Physiol..

[15] Daida A., Kurotani T., Yamaguchi K., Takahashi Y., Ichinohe N. (2024). Different numbers of conjunctive stimuli induce LTP or LTD in mouse cerebellar Purkinje cell. Cerebellum.

[16] Karachot L., Kado R.T., Ito M. (1994). Stimulus parameters for induction of long-term depression in in vitro rat Purkinje cells. Neurosci. Res..

[17] Schreurs B.G., Oh M.M., Alkon D.L. (1996). Pairing-specific long-term depression of Purkinje cell excitatory postsynaptic potentials results from a classical conditioning procedure in the rabbit cerebellar slice. J. Neurophysiol..

[18] Suvrathan A., Raymond J.L. (2018). Depressed by learning-heterogeneity of the plasticity rules at parallel fiber synapses onto purkinje cells. Cerebellum.

[19] Wetmore D.Z., Jirenhed D.A., Rasmussen A., Johansson F., Schnitzer M.J., Hesslow G. (2014). Bidirectional plasticity of Purkinje cells matches temporal features of learning. J. Neurosci..

[20] Rasmussen A., Jirenhed D.A., Zucca R., Johansson F., Svensson P., Hesslow G. (2013). Number of spikes in climbing fibers determines the direction of cerebellar learning. J. Neurosci..

[21] Lippiello P., Hoxha E., Tempia F., Miniaci M.C. (2020). GIRK1-Mediated inwardly rectifying potassium current is a candidate mechanism behind Purkinje cell excitability, plasticity, and neuromodulation. Cerebellum.

[22] Ramu Y., Xu Y., Lu Z. (2008). Engineered specific and high-affinity inhibitor for a subtype of inward-rectifier K+ channels. Proc. Natl. Acad. Sci. U. S. A..

[23] Johansson F., Hesslow G. (2020). Kir3 channel blockade in the cerebellar cortex suppresses performance of classically conditioned Purkinje cell responses. Sci. Rep..

[24] Jirenhed D.A., Bengtsson F., Hesslow G. (2007). Acquisition, extinction, and reacquisition of a cerebellar cortical memory trace. J. Neurosci..

[25] Bouton M.E., Sunsay C. (2003). Importance of trials versus accumulating time across trials in partially reinforced appetitive conditioning. J. Exp. Psychol. Anim. Behav. Process..

[26] Vardaris R.M., Fitzgerald R.D. (1969). Effects of partial reinforcement on a classically conditioned eyeblink response in dogs. J. Comp. Physiol. Psychol..

